# The Harms of Enhancement and the Conclusive Reasons View

**DOI:** 10.1017/S0963180114000218

**Published:** 2015-01

**Authors:** THOMAS DOUGLAS

**Keywords:** enhancement, Allen Buchanan, harm, justice, fairness, precautionary principle

## Abstract

Many critics of bioenhancement go to considerable lengths to establish the existence of reasons against pursuing bioenhancements but do little to establish the absence of reasons in favor. This suggests that they accept what Allen Buchanan has called the conclusive reasons view (CRV). According to this view, our reasons against bioenhancement are obviously decisive, so there is no need to balance them against countervailing reasons. Buchanan criticizes the CRV by showing that the reasons most commonly adduced against bioenhancement are not decisive, or, at least, not obviously so.

In this article, I suggest that both Buchanan and the authors to whom he is responding underestimate the strength of the case for the CRV. There are, I argue, harm-based reasons against bioenhancement that provide stronger support to the CRV than the reasons that have most often been adduced by critics of enhancement. However, I then argue that even these harm-based reasons are not obviously decisive. Thus, I ultimately agree with Buchanan about the falsity of the CRV, though I disagree with him about the reasons for its falsity.

Allen Buchanan has recently argued that, at least in liberal societies, political institutions should treat bioenhancement—the use of biotechnologies to augment the capacities of already healthy, normal people—as a legitimate enterprise.^1,2,3^ That is to say, they should (1) allow individuals and organizations “considerable freedom” to develop and use bioenhancement technologies, (2) devote “significant public resources” to research expected to produce them, and ( 3) promote debate about, and sound policies on, their use.^4^

In defending this view, Buchanan takes himself to be arguing against the views taken by so-called bioconservative authors such as Francis Fukuyama, Leon Kass, and Michael Sandel.^5,6,7,8,9^ Though these authors have not been entirely clear about what stance political institutions should take toward bioenhancement, they do appear to be committed to the view that bioenhancement ought not to be treated as a legitimate enterprise, in Buchanan’s sense. For example, Michael Sandel portrays himself as offering an “argument against enhancement” not further specified,^10^ and Francis Fukuyama urges that we protect “the full range of our complex, evolved natures against attempts at self-modification”^11^ These claims are naturally read as favoring a model in which political institutions generally prohibit or discourage bioenhancements. In what follows, I assume that Fukuyama, Kass, and Sandel indeed take their arguments to show that bioenhancement should not be treated as a legitimate enterprise. I assume, as I will henceforth put it, that they are arguing against *legitimating* bioenhancement.

It is notable that Fukuyama, Kass, and Sandel do not engage in a balancing of the pros and cons of legitimating enhancement. Rather, they lay out one or a few reasons against such legitimation. For example, Michael Sandel bases his case against legitimation almost exclusively on the claim that engaging in bioenhancement expresses an objectionable attitude—an attitude of “mastery” toward oneself. He does discuss other general arguments that have been offered against enhancement, but he dismisses them as inadequate.^12^ Kass and Fukuyama both endorse a broader range of concerns about bioenhancement. But, like Sandel, they engage in no attempt to weigh these concerns against possible upsides of legitimating bioenhancement.

Buchanan takes this to indicate that these bioconservative authors accept the conclusive reasons view (CRV), which we can understand as the view that those who design and uphold political institutions (henceforth simply “political agents”) have conclusive reasons not to legitimate bioenhancement.^13^ Conclusive reasons are reasons that are obviously decisive. They are decisive in the sense that they alone outweigh all countervailing reasons. And they are obviously so in the sense that their decisiveness is clear in advance of engaging in any explicit weighing against countervailing reasons. Unless the CRV is attributed to Fukuyama, Kass, and Sandel, it is difficult to make sense, in any charitable way, of their tendency to evade any balancing of the pros and cons of enhancement.^14^

As Buchanan frames the debate, then, the interesting question is whether bioconservatives such as Fukuyama, Kass, and Sandel have successfully defended the CRV. Buchanan argues that they have not. The primary reasons against legitimating bioenhancement invoked by Fukuyama, Kass, and Sandel are that bioenhancements

1) Are unnatural
2) Will compromise or offend against human nature
3) Will alienate us from our authentic selves
4) Express a lack of gratitude and an attitude of mastery

But Buchanan argues that none of these considerations constitutes a conclusive reason against legitimating bioenhancement.^15^

I believe that Buchanan’s arguments on this front are persuasive. One might wonder, however, whether he and his opponents have considered the strongest case for the CRV. It is questionable whether reasons 1–4 were ever promising candidates as conclusive reasons, for none of these considerations clearly appeals to *harm to others.* None clearly indicates that voluntarily engaging in bioenhancement will cause harm to anyone other than the individual who pursues the enhancement. The concern that enhancement might render the enhanced individual inauthentic could be construed as a concern about one way in which engaging in enhancement might harm *oneself*, for it might be thought that authenticity contributes to individual well-being. Similarly, insofar as retaining our human nature contributes to our well-being, the view that enhancement might compromise human nature could perhaps be construed as a concern about harm to self. However, neither the appeal to authenticity nor the appeal to human nature points clearly to any harm that one person’s pursuit of bioenhancement might impose on *others*. And the concerns about unnaturalness and the expression of objectionable attitudes arguably do not point to harms at all. The former is normally understood as an objection to the *means* of biomedical enhancement and the latter as an objection to the *motives* for which it would be pursued, whereas harm to others is an *effect*.^16^

The absence of any appeal to harm to others is problematic because it is arguably a fundamental and plausible tenet of liberalism that a voluntary practice should be treated as legitimate *unless* it causes harm to others. Of course, bioconservative writers well might reject liberalism or, at least, this tenet of it.^17^ Nevertheless, the liberal tenet is plausible, and if arguments against legitimating enhancement rely on its falsity, this will at least tend to diminish the attractiveness of those arguments.

It may, however, be possible to defend the CRV in a way that is consistent with the aforementioned liberal thesis: one might appeal to ways in which voluntary pursuit of enhancement by some might inflict harm on others. Opponents of bioenhancement have, as we will see, pointed out various ways in which bioenhancements undergone by some individuals might harm others. But they have not, to my knowledge, sought to assemble these into a systematic argument for the CRV. My question, in the remainder of this article, is, “Do concerns about harm to others give us conclusive reasons not to legitimate bioenhancement?” I begin by outlining five ways in which enhancement might cause harm to others. I then consider whether these can ground an argument for the CRV.

## Five Ways in Which Enhancement by Some Might Harm Others

### Deliberate Harmful Use

One way in which bioenhancement could cause harm to others is by increasing the effectiveness or efficiency of those engaged in deliberately harmful activities. The clearest example of this is probably bioenhancement in the military. Modafinil is a drug thought to increase the ability to function when deprived of sleep in some circumstances, and it has been approved for use by the U.S. Air Force to help soldiers and pilots fight when sleep deprived.^18^ Because one of the aims of military combat is typically to inflict harm on one’s opponents, one might expect that, where modafinil is effective at increasing combative effectiveness, it will tend to increase the amount of harm inflicted on those opponents.

### Competitive Effects

A second way in which bioenhancement could cause harm to others is by increasing the effectiveness of enhanced individuals in some competitive activity, thereby placing the unenhanced at a competitive disadvantage. This is probably the most frequently mentioned harm of bioenhancement and has been widely adduced in support of restrictive approaches to it.^19,20,21,22^ The classic examples come from sports; if one athlete uses performance-enhancing biomedical technologies, she clearly places her competitors at a competitive disadvantage. However, similar concerns can also be raised about cognitive enhancements insofar as they are used by students preparing for exams or anyone else engaged in competitive, cognitively demanding activities.

### Contribution to Coercive Enhancement

Another commonly mentioned way in which voluntary bioenhancement might lead to harm to others is by causally contributing to subsequent coercive bioenhancements, which might be thought harmful insofar as they impinge on individual autonomy.^23,24,25,26^ There are two distinct ways in which one person’s voluntary enhancement might lead others to be coerced into unwanted enhancements. First, one person’s voluntary enhancement might increase the competitive pressure on others to follow suit: the unenhanced may need to engage in enhancements to maintain their competitiveness with enhanced individuals, and thus to maintain their status quo ante levels of well-being. The initial enhancement thus puts pressure on others to enhance, and this might be thought to amount to a soft form of coercion that somewhat interferes with autonomy. Second, voluntary enhancement by some might lead to straightforwardly coercive enhancements by breaking down antienhancement attitudes and conventions, perhaps ultimately leading to a society in which governments or others feel free to make enhancements legally obligatory. For example, suppose a number of individuals engage in bioenhancements that dramatically enhance their economic productivity across a range of occupations. One can imagine that, observing this effect, a government might be tempted to make the bioenhancement compulsory. After all, belief in the productivity-increasing effects of primary education played an important role in moves to make it compulsory.^27^

### Undermining Harm Aversion

A less frequently discussed way in which enhancement might cause harm is by undermining those psychological resources that typically hold people back from harming others. These might include empathic ability, feelings of sympathy, and the capacity for moral reasoning. We can imagine various ways in which enhancements might weaken these resources. One possibility is that aggressive pursuit of enhancement by some individuals might confer on those individuals capacities so different from those possessed by others that the enhanced can no longer empathize or sympathize with the unenhanced.^28^ Another possibility is that enhancements might more directly reduce the psychological costs of harming others. Think of an intervention that enhances forgetfulness in soldiers, thus allowing them to commit atrocities over and over without succumbing to posttraumatic stress disorder. Or consider a ruthless businessman who seeks to enhance his efficiency by biomedically suppressing feelings of altruism. These enhancements could surely increase the prevalence of harmful behavior.

### Increasing Liability to Permissible Harm

A fifth possibility that has been considered by a number of authors on either side of the enhancement debate is that bioenhancements undergone by some people might inflict a kind of metaharm on those who remain unenhanced: they might increase the range of circumstances in which the unenhanced can be permissibly harmed.^29,30,31,32,33,34,35^

To see how this could occur, note that cognitively normal adult humans are usually thought to have the right to exclude children and cognitively disabled adults from effective political participation by introducing political arrangements that are much too complex for them to effectively participate in, and that are, in some cases, entirely closed to their participation. For example, we have the right to introduce democratic institutions that some cognitively disabled adults cannot understand, and in which children are legally prevented from participating. Now suppose that there existed superenhanced beings capable of much more sophisticated forms of social and political coordination than us. It might be thought that they would have the right to introduce more sophisticated sociopolitical arrangements at the expense of ours, even though we would then be excluded from effective engagement in the dominant cooperative system.^36^ There would thus be a sense in which the existence of the superenhanced beings would have rendered ordinary humans more liable to permissible harm of a certain kind—the harm of being excluded from political participation. And this increase in liability might itself be regarded as a harm.

## Are There Conclusive Harm-Based Reasons against Legitimating Bioenhancement?

There are, then, several ways in which bioenhancements undergone by some individuals could impose harms on others. Moreover, we might expect that at least some actual bioenhancements will indeed have these harmful consequences. And we might reasonably suppose that treating bioenhancement as a legitimate enterprise would, by increasing the overall amount of bioenhancement that takes place, tend to increase the frequency with which these harms would occur. Do harm-based considerations thus give us conclusive reasons not to legitimate bioenhancement?

One reason to doubt that they do is that it seems doubtful whether *all* bioenhancements would cause harm to others. If only some would do so, then it might be acceptable to legitimate bioenhancement. Recall that political institutions legitimate bioenhancement if and only if they (1) allow individuals and organizations “considerable freedom” to develop and use bioenhancement technologies (2) devote “significant public resources” to research expected to produce them, and (3) promote debate about—and sound policies on—their use. This is consistent with prohibiting or discouraging *some* bioenhancements. Perhaps, then, the right approach would be to legitimate bioenhancement but prohibit or discourage specific types of bioenhancement on harm-based grounds.

Another reason to doubt whether considerations of harm give us conclusive reasons not to legitimate bioenhancement is that there may be some harms that we have no reason to avoid. For instance, I noted previously that bioenhancements might harm others through competitive effects. One person’s bioenhancement might allow her to compete more effectively with others, thus harming those others. But it is not clear that we have reasons to avoid imposing all competitive harms. Suppose that Jane is at a competitive disadvantage to her classmates, in terms of academic performance, because, unlike them, she is unable to afford the latest textbook. However, suppose that she does have access to cognitive-enhancing drugs that others are not using. By taking these drugs, she would harm others, making those others less effective competitors than they would otherwise have been. However, because it is plausible that those others currently enjoy an unfair competitive advantage, it is not obvious that she has any reason to abstain from imposing this harm.

For the sake of argument, however, let us suppose that either (1) *all* bioenhancements would result in harm to others, including harm that there is reason to avoid, or (2) *many* bioenhancements would result in harm to others, including harm that there is reason to avoid, *and* there is no effective form of regulation that would prevent these bioenhancements while enabling others. If either of these assumptions is correct, then harm-based considerations would, I take it, give us some reason not to legitimate bioenhancement. I now turn to consider whether these reasons count *conclusively* against legitimating bioenhancement.

## Harms versus Benefits

One obvious problem with the suggestion that the aforementioned harm-based considerations constitute conclusive arguments against bioenhancement is that bioenhancements can *benefit* others as well as harming them. For example, Buchanan argues that, like nonbiomedical enhancements, such as education and information technology, many bioenhancements should be expected to significantly increase human productivity—our ability to produce things we value with the resources we have.^37^ As well as benefiting the enhanced, this is predicted to have spillover benefits for the unenhanced, for example, by lowering prices, accelerating scientific progress, and assisting the mitigation of global threats such as pandemics and climate change.^38,39^ It seems possible that reasons to bring about these benefits by legitimating bioenhancement would outweigh reasons to prevent harms by not doing so. Certainly, many of us would be inclined to say that our predecessors had decisive reasons to bring about the great historical nonbiomedical enhancements, such as the development of written language and schools, even though these enhancements also caused both harms and benefits.

At this point, there seem to be three main routes open to the proponent of the CRV. One would be to argue, perhaps by appealing to a strong variant of the precautionary principle, that when a course of action is associated with serious harm, one ought not to pursue it, regardless of the benefits. Another would be to argue that the benefits of enhancement are qualitatively different from, and less important than, the harms. Finally, a third response would be to argue that the benefits of enhancement will be smaller in magnitude than the harms. In what follows I consider whether any of these approaches establishes that harm-based reasons against legitimating bioenhancement are conclusive—that is, *decisive*, and obviously so, even in advance of any weighing against countervailing considerations.

## The Precautionary Principle

The precautionary principle was developed in northern Europe in the late 1960s and is frequently advocated as a guide for assessing projects that pose environmental risks. It has been formulated in many different ways.^40^ Perhaps the two most frequently discussed variants hold, respectively, that, in assessing the risk-benefit balance posed by some policy or project,

1) Lack of certainty about possible risks should not prevent those risks from being taken into account.^41^
2) The burden of proof is on those who claim that a risky policy or project should be pursued.^42^

Neither of these variants of the principle are of much help to the proponent of the CRV. These formulations *do* bear on how the risk-benefit balance associated with legitimating bioenhancement should be determined. But once we have established that there are both risks of harm and prospects of benefit associated with legitimating bioenhancement—as, plausibly, we already have—these variants of the precautionary principle lose relevance, for they tell us nothing about how one should respond to a given risk-benefit profile.

A third variant of the precautionary principle appears more promising as a potential basis for the CRV. This variant holds that

3) When a project or policy is associated with a serious risk, it should not be pursued, regardless of its expected benefits (the strong precautionary principle).^43^

The seriousness of the risk would typically be determined by the severity of the bad outcome that may occur, though the likelihood and certainty of that outcome might also be relevant. If this variant of the precautionary principle is correct, and if the risks of harm posed by legitimating bioenhancement are serious, then we would have decisive harm-based reasons not to legitimate bioenhancement. Moreover, the decisiveness of those reasons could be established without weighing them against any benefits: the existence of a serious risk of harm combined with acceptance of the strong precautionary principle is sufficient to rule out the legitimation of bioenhancement.

The strong precautionary principle is, however, susceptible to a devastating objection that has been advanced, in different forms, by Neil Manson and Cass Sunstein.^44,45^ Suppose that we are considering whether to adopt some policy *P*, and we wish to apply the strong precautionary principle. There are two different ways in which we might apply it. One option would be to simply assess the likely risks of *P*, determine whether any are serious, and, if they are, conclude that *P* should not be adopted. But suppose that the following situation obtains: *P* will create some serious risks, but any alternative policy (including the status quo policy) is associated with even more serious risks. In this case, considerations of precaution should count in favor of *P*. Yet if we apply the strong precautionary principle in the way I have just suggested, it will instead count against *P*. It will instruct us not to adopt *P.* The problem arises because the risk associated with alternatives to *P* is ignored. This suggests an alternative, more comprehensive approach in which we apply the principle to *P* and *all alternative policies* (including the status quo policy). For each alternative, we determine whether it poses a serious risk, and, if it does, we conclude that it should not be adopted. But if we use this method, the strong precautionary principle may imply that none of the available alternatives should be adopted, because each may pose a serious risk. In this case the principle provides guidance that cannot be followed, because it is clearly impossible to reject all policy alternatives. Thus, if the strong precautionary principle is applied in a restricted way, it may give the wrong guidance, and if it is applied in a comprehensive way, it may give no practical guidance at all.

It might be thought that we should nevertheless apply the strong precautionary principle in cases in which it *can* be applied comprehensively and still yield guidance that can be followed—that is, in cases where some *but not all* alternatives pose a serious risk of harm. However, it seems unlikely that this is the case when the decision is between legitimating and not legitimating bioenhancement. This is because both legitimating and not legitimating bioenhancement are likely to be associated with serious risks. We have already discussed the risks of harm associated with legitimating bioenhancement. Risks associated with not legitimating bioenhancement might include the risk that, in the absence of widespread enhancement, we will fail to solve major global problems such as climate change before they wreak great havoc. They might also include a risk that, in the absence of state legitimation of bioenhancements, they will be pursued underground without proper safeguards and thus potentially in ways that will cause significant harm, for example, through medical side effects. It seems likely that the strong precautionary principle will advise against *not* legitimating bioenhancement as well as against legitimating it.

At this point, we could weaken the strong precautionary principle to something like the following:

4) In deciding between alternative policies, we should attach greater weight to risks associated with each policy than to the benefits (the weak precautionary principle).

This principle may well yield practical guidance on the question of whether to legitimate bioenhancement: it will not rule out all available courses of action. But it faces further problems. For example, it relies on there being a meaningful distinction between risks and the loss of benefits, but it is not clear that there is. Suppose we choose not to legitimate bioenhancement and thereby sacrifice certain productivity benefits that would otherwise have been obtained. One could argue that this loss of benefits should itself qualify as a risk. Another problem is that it remains unclear *why* risks should be given more weight than benefits.

Moreover, even if the weak precautionary principle is plausible, it is far from clear that it could support the CRV. According to this principle, in deciding whether to legitimate bioenhancement, we should give *some* weight to the benefits of doing so, even though we should give more weight to the risks. But it seems possible that the benefits of legitimating enhancement would be substantially greater in magnitude than the risks, and if this is so, then even if risks should be given more weight than the benefits, the benefits might, in this case, carry the day. Thus, even if we accept the weak precautionary principle, it will not be *obvious* that our risk-based reasons against legitimating bioenhancement are decisive.

## Qualitative Differences

Given the problems faced by an attempt to justify the CRV through appeal to the precautionary principle, it seems wise to look elsewhere for a defense of that view.

One possible defense would maintain that the suggested benefits of bioenhancement, in the form of increased productivity, are qualitatively less important than the harms. For example, it might be argued that at least some of the harms of enhancement would be a matter of *justice* or *rights*, whereas the benefits would not. (I henceforth pursue this suggestion using the language of justice, though, given that there is plausibly a close connection between justice and rights, I suspect what I say could be translated into the language of rights.)

When a soldier waging an unjust war undergoes an enhancement that increases his efficiency, this arguably contributes to not only the imposition of harms but also to the *unjust* imposition of harms (henceforth simply ‘injustice’). Similarly, if voluntary enhancement by some encouraged the state to subsequently pursue coercive enhancements, it might be thought that the enhancements would have contributed to injustice, for it could be unjust for the state to coerce people to undergo bioenhancements. These cases suggest that legitimating enhancement could facilitate injustice, and this might support the view that political agents have reasons *of justice* not to legitimate bioenhancement. Presumably, the most powerful reasons of justice are reasons not to unjustly inflict harm on oneself. But it might be argued that political agents also have reasons of justice not to facilitate the unjust imposition of harm by others.

On the other hand, it is, perhaps, less clear that political agents have reasons of justice to bring about productivity benefits through legitimating bioenhancement. Arguably, were they to forego these benefits, they would be neither unjustly harming anyone nor facilitating unjust conduct by others. They would, of course, be failing to realize certain benefits, but many would doubt that justice requires political agents to realize such benefits.

If it is correct that there are reasons of justice against legitimating bioenhancement but no reasons of justice in its favor, then the CRV will look quite plausible. This is because justice is plausibly a moral consideration of overriding importance.^46^

It is, however, doubtful whether considerations of justice count only against, and never for, bioenhancement. This is because bioenhancements could have other benefits, besides those of increased productivity, and some of these might well be a matter of justice. That is to say, bioenhancements may have benefits that political agents have reasons of justice to promote. If so, there will be considerations of justice on both sides of the ledger.

### Preventing Injustice through Bioenhancement

How might considerations of justice support bioenhancement? An initial possibility is that bioenhancements might alter the enhanced individual’s moral psychology in a way that helps to prevent her from unjustly harming others.^47^ There is already at least one biomedical intervention that is regularly used in part to prevent injustice. Antiandrogenic drugs are used in several jurisdictions to prevent recidivism in sex offenders, a practice that has become known as “chemical castration.” Though the evidence is not currently conclusive, chemical castration is thought to reduce rates of reoffending in certain classes of sex offenders, including some pedophiles.^48^ It is unclear whether this intervention should be regarded as a bio*enhancement*, because it is being used to correct what is clearly an abnormality (if not a disease). However, the existence of biomedical interventions that appear to be capable of reducing unjust conduct in certain abnormal individuals at least raises the prospect that it might be possible to develop biomedical interventions that also reduce unjust conduct in normal individuals. Impulsive violent aggression is arguably a normal behavior in some demographic groups, but it can unjustly impose harm. We might expect that, in the future, biomedical interventions will be capable of attenuating the disposition to harm others through impulsive violence. Indeed, some drugs have already shown promise in attenuating this disposition in certain groups.^49,50^

Further support for the hypothesis that bioenhancements could attenuate the risk of unjust conduct comes from studies of biological influences on fairness-related behavior. For instance, an oft-cited Israeli study found that judges were substantially more likely to make strict parole decisions if more time had elapsed since their last food break.^51^ Imposing *overly* strict parole decisions can plausibly constitute an injustice in some cases. Thus, the Israeli study could be read as suggesting that the time since a judge’s last meal break influences the risk that a judge will unjustly harm an offender. Though the researchers were not able to determine the mechanism of the effect, it is likely that it was in part a biological effect of the judges’ food intake. But if food intake can biologically influence a judge’s disposition to unjustly harm an offender, it is surely plausible that a biomedical intervention, such as a drug, could do the same.^52^ This suggestion receives further support from recent work showing that dietary interventions that manipulate brain serotonin activity can influence fairness and punishment-related behaviors under laboratory conditions.^53^

### Correcting Past Injustices through Bioenhancement

Another way in which bioenhancement might have benefits that are a matter of justice is that they may be used in ways that correct, or partially correct, past injustices. This possibility can be illustrated straightforwardly with the aid of hypothetical cases. Consider, first, this case:

The adult members of a minority group were, as children, unjustly excluded from the education available to others by a racist government. As a result, they compete less successfully in the labor market than their contemporaries from other ethnic groups. A new, more enlightened government now in power decides to provide intensive adult education programs for members of the minority. As a result of engaging in these programs, many members of the minority group are able to compete more successfully with their contemporaries.

I think most would agree that the educational program offered by the government in this case helps to correct a past injustice perpetrated by the government.

But now consider a second case in which everything is the same as before, except that this time the educational deficit is too severe to be much altered by an education program alone. So, instead, the government decides to offer an intensive education program *plus* a cognitive-enhancing drug that improves learning ability. This program substantially increases the success of those who undergo it in the labor market.

It seems clear that if the education program in the first case helped to correct an injustice, then the combined education-bioenhancement program in the second case does so too. Moreover, the bioenhancement described here might well become technologically feasible. There are already drugs available that augment various aspects of cognitive function, including working memory and attention,^54^ and, though the long-term effects of these drugs on learning in normal individuals has not been investigated, it would not be surprising if they turned out to be positive.

### Justice on Both Sides

Given the possibilities just described, it seems that both those who oppose and those who support legitimating bioenhancement can appeal to considerations of justice. I have granted that legitimating bioenhancements might contribute to injustice—that is, the unjust imposition of harm. But I have also argued that bioenhancements could *prevent* or *correct* such injustice. Thus, *not* legitimating enhancement may also contribute to injustice, or to the persistence of unjustly inflicted harms—it might do this by preventing these preventative or corrective bioenhancements from taking place. Considerations of justice—arguably the most important moral considerations—can thus be found on both sides of the ledger. This casts doubt on the suggestion that considerations of justice could be invoked in support of the CRV.

It might be argued, at this point, that the justice-based reasons *against* legitimating bioenhancement are of a more powerful variety than the justice-based reasons *for* doing so. Arguably, by legitimating bioenhancement political agents would be *actively* contributing to future injustice, whereas by declining to legitimate bioenhancement they would merely be passively allowing some future injustice to occur, and some unjustly inflicted harms to persist, uncorrected. It might be held that there are stronger reasons not to positively contribute to injustice than to prevent or correct it.

However, this response relies on the view that to legitimate bioenhancement is to take an active step in a way that to decline to legitimate it is not. This, I think, is questionable, for at least two reasons. First, at least on liberal accounts of political morality, the recommended default position is normally to legitimate any voluntary activity. On these accounts, there is a sense in which *not* legitimating bioenhancement is in fact more active than is legitimating it: the former involves deviation from the moral default position, whereas the latter does not. Second, it seems fair to say that, at present, most liberal democracies treat some bioenhancements as legitimate and others as not legitimate. For example, almost all bioenhancements that enhance sporting performance are widely prohibited: bioenhancement in sports is certainly not treated as a legitimate enterprise in Buchanan’s sense. On the other hand, cosmetic procedures intended to improve on normal appearance generally *are* treated as legitimate. Thus, both legitimating bioenhancement and not legitimating it would require some change from the status quo. In a sense, then, both would involve taking active steps. Arguably, then, political agents face a choice between actively contributing to injustice through legitimating enhancement and actively contributing to injustice through not legitimating it.

## Quantitative Differences

A third and final way of defending the view that considerations of harm provide conclusive reasons against legitimating bioenhancement would appeal to a quantitative balancing of harms and benefits. One might maintain that the harms associated with legitimating bioenhancement are likely to exceed the benefits in magnitude. Or, if one believes that considerations of justice serve as trump values, one might claim that legitimating bioenhancement will produce more serious injustice than it corrects or prevents. For example, one might argue that, although it is *possible* that bioenhancements might be used in ways that prevent or correct injustice, they would only very rarely be used in these ways. Much more frequently, they will be used in ways that contribute to injustice.

I suspect that a quantitative argument of this kind will be the most promising argument against legitimating bioenhancement. Still, it is doubtful that it can support the conclusive reasons view. Quantitative considerations will give us *conclusive* reasons not to legitimate bioenhancement only if (1) it is *obvious* that legitimating enhancement will produce more harm than benefits (or more injustice than it prevents or corrects) and (2) this constitutes a decisive reason not to legitimate enhancement. But requirement 1 is not satisfied. Were we to attempt to predict and weigh the harms and benefits of legitimating bioenhancement, we might ultimately be able to arrive at the conclusion that the relevant harms will indeed outweigh the relevant benefits. But it is difficult to see how we could be justified in concluding this in the absence of any such weighing.

Admittedly, one can imagine circumstances in which we would, perhaps, be justified in concluding that the relevant harms are likely to outweigh the relevant benefits, even in advance of such weighing. Perhaps one indicator of how future bioenhancements are likely to be used is how existing nonbiomedical enhancements have been used. We might regard computers, the Internet, and telephones as nonbiomedical enhancement technologies: they augment our communication abilities, among others. Perhaps we could also regard certain institutions as nonbiomedical enhancements: schools and universities arguably serve as cognitive enhancements, whereas the criminal justice system could be thought of as a kind of behavioral enhancement. If these existing nonbiomedical enhancements had clearly and overwhelmingly been used in harmful rather than beneficial ways, this could give us strong reason to believe that future *bio*enhancements will also be used in ways that produce more harm than benefit. Perhaps it would even make it obvious that this is likely to be so. But this is not, or at least not clearly, our current situation. Though some might maintain that existing nonbiomedical enhancements have been more harmful than beneficial, this would be a highly contentious position. It is certainly not *clearly* the case. At most, then, the ways in which existing nonbiomedical enhancements have been used provide weak and uncertain support for the view that legitimating bioenhancement will produce more harm than benefit. It is difficult to see how such support could make the view obviously correct.

Precisely parallel thoughts apply to the justice-based variant of the quantitative argument. Though some might argue that existing nonbiomedical enhancements have produced more injustice than they have prevented or corrected, this would be a contentious position. It is certainly not *clearly* the case. Thus, it is difficult to see how past experiences with non-biomedical enhancements could make it *obvious* that legitimating biomedical enhancement will produce more injustice than it prevents or corrects.

## Conclusion

The conclusive reasons view maintains that we have conclusive reasons not to legitimate bioenhancement—reasons that are decisive, and whose decisiveness is already obvious. In this article, I have considered whether considerations of harm might support this view. I first identified five ways in which bioenhancements might impose such harms and then distinguished three ways in which one might argue that these reasons count conclusively against legitimating bioenhancement: by appealing to the precautionary principle; by arguing that the relevant harms are qualitatively different from, and more important than, the benefits of bioenhancement; and by arguing that the harms exceed the benefits in magnitude. However, I argued that none of these arguments are able to sustain the conclusive reasons view. It may well turn out that one or more of the reasons invoked by these arguments constitute a decisive reason not to legitimate bioenhancement. But this is not obvious in advance of weighing these reasons against countervailing ones. This is a weighing that opponents of bioenhancement have yet to engage in.

## Notes

1. BuchananAE Enhancement and the ethics of development. Kennedy Institute of Ethics Journal2008;18(1):1–34.1856157610.1353/ken.0.0003

2. BuchananAE Beyond Humanity? The Ethics of Biomedical Enhancement. Oxford: Oxford University Press; 2011.

3. BuchananAE Better Than Human: The Promise and Perils of Enhancing Ourselves. New York: Oxford University Press; 2011.

4. See note 2, Buchanan 2011, at 16.

5. See, for example, FukuyamaF Our Posthuman Future: Consequences of the Biotechnology Revolution. New York: Profile Books; 2002.

6. FukuyamaF Transhumanism. Foreign Policy2004;144:42–3.

7. KassLR Life, Liberty, and the Defense of Dignity. San Francisco: Encounter Books; 2002.

8. KassLR Ageless bodies, happy souls. The New Atlantis2003;1:9–28.15584192

9. SandelM The Case against Perfection: Ethics in the Age of Genetic Engineering. Cambridge, MA: Harvard University Press; 2007.

10. See note 9, Sandel 2007, at 92, 95.

11. See note 5, Fukuyama 2002, at 172.

12. See note 9, Sandel 2007, at 5–24.

13. See note 1, Buchanan 2008, and note 2, Buchanan 2011.

14. See note 1, Buchanan 2008, at 19–20.

15. See note 1, Buchanan 2008.

16. It is true that Michael Sandel, the chief exponent of the concern about expressing objectionable attitudes, connects this concern with a concern about the undermining of social solidarity, which might be thought to be ultimately a concern about harm to others (see note 9, Sandel 2007, at 89–92). However, I am inclined to think that he conflates two distinct arguments here. Taken at face value, the concern about expressing objectionable attitudes is not a consequence-based concern; it is a concern about motives. By contrast, the concern about undermining social solidarity plainly is a concern about the possible consequences of biomedical enhancement. It is, of course, possible that the motives to which Sandel objects would causally contribute to the undermining of social solidarity. In that case, the appeal to objectionable attitudes could be understood as a disguised appeal to a consequence-based concern. However, there is little reason to interpret Sandel’s objection in this way: he does not provide any argument for such a causal connection, and he is elsewhere at pains to emphasize that his appeal to objectionable attitudes is *not* a consequence-based concern (e.g., at 95–96). This leads me to conclude that Sandel’s appeal to the undermining of social solidarity is distinct from his main, attitudes-based critique of enhancement. I consider an argument similar to his solidarity-based critique later in this article.

17. Michael Sandel suggests that standard liberal principles are unable to capture the problematic nature of bioenhancement and takes this to count not in favor of bioenhancement but against liberalism. See note 9, Sandel 2007, at chap. 1.

18. CaldwellJA, CaldwellJL Fatigue in military aviation: An overview of U.S. military-approved pharmacological countermeasures. Aviation, Space, and Environmental Medicine2005;76(1 Suppl):C39–C51.16018329

19. Academy of Medical Sciences. Brain Science, Addiction and Drugs. London: Academy of Medical Sciences; 2008, at 158–9.

20. ChatterjeeA A medical view of potential adverse effects. Nature2009;457(7229):532–3.1917710410.1038/457532c

21. McKibben B. Designer genes. *Orion* 2003 Apr 30; available at http://www.orionmagazine.org/index.php/articles/article/119 (last accessed 15 Sep 2014).

22. President’s Council on Bioethics. *Beyond Therapy: Biotechnology and the Pursuit of Happiness*. Washington, DC: President’s Council on Bioethics; 2003, at 131–4, 280–1.

23. See note 22, President’s Council 2003, at 135–7, 283–5.

24. FarahMJ, IllesJ, Cook-DeeganR, GardnerH, KandelE, KingP, et al Neurocognitive enhancement: What can we do and what should we do?Nature Reviews Neuroscience2004;5(5):421–5.10.1038/nrn139015100724

25. British Medical Association. Boosting Your Brain Power: Ethical Aspects of Cognitive Enhancements. London: British Medical Association; 2007, at 19–23.

26. See note 9, Sandel 2007, at 18–19.

27. For example, the Elementary Education Act of 1870, which paved the way to compulsory primary education in England and Wales, was motivated largely by a concern for those countries to maintain their international economic competitiveness. See RamirezFO, BoliJ The political construction of mass schooling: European origins and worldwide institutionalization. Sociology of Education1987;60(1):2–17.

28. See note 9, Sandel 2007, at 89–91.

29. Wikler DI. Paternalism in the age of cognitive enhancement: Do civil liberties presuppose roughly equal mental ability. In: Savulescu J, Bostrom N, eds. Oxford: Oxford University Press; 2009;341–55.

30. WilsonJ Transhumanism and moral equality. Bioethics2007;21(8):419–25.1784544810.1111/j.1467-8519.2007.00579.x

31. BuchananA Moral status and human enhancement. Philosophy & Public Affairs2009;37(4):346–81.

32. See note 2, Buchanan 2011.

33. DouglasT Human enhancement and supra-personal moral status. Philosophical Studies2013;162(3):473–97.2357682110.1007/s11098-011-9778-2PMC3617590

34. DeGraziaD Genetic enhancement, post-persons and moral status: A reply to Buchanan. Journal of Medical Ethics2012;38(3):135–9.2207477110.1136/medethics-2011-100126

35. AgarN Why is it possible to enhance moral status and why doing so is wrong?Journal of Medical Ethics2013;39(2):67–74.2292343910.1136/medethics-2012-100597

36. This example is modified from Wikler 2009 (see note 29) and Buchanan 2009 (see note 31).

37. See note 1, Buchanan 2008, and note 2, Buchanan 2011, at 35–67.

38. BostromN, OrdT The reversal test: Eliminating status quo bias in applied ethics. Ethics2006;116(4):669–70.10.1086/50523317039628

39. See also note 2, Buchanan 2011, at 38–49, though compare PerssonI, SavulescuJ Unfit for the Future: The Need for Moral Enhancement. Oxford: Oxford University Press; 2012.

40. SunsteinCR Laws of Fear: Beyond the Precautionary Principle. Cambridge: Cambridge University Press; 2005, at 18.

41. United Nations Environment Programme. *Rio Declaration on Environment and Development*. Nairobi: United Nations Environment Programme; 1992, principle 15.

42. Ashford N, Barrett K, Bernstein A, Constanza R, Costner P, Cranor C, et al. *Wingspread Statement on the Precautionary Principle*; 1998; available at www.gdrc.org/u-gov/precaution-3.html (last accessed 15 Sep 2014).

43. See, for a similar formulation of the precautionary principle, Seas at Risk. *Final Declaration of the First European “Seas at Risk” Conference (Copenhagen, 26–28 October 1994): A Shadow Declaration*. Brussels: Seas at Risk; 1994, at 28: “If the ‘worst case scenario’ for a certain activity is serious enough then even a small amount of doubt as to the safety of that activity is sufficient to stop it taking place.”

44. MansonNA Formulating the precautionary principle. Environmental Ethics2002;24:263–74.

45. See note 40, Sunstein 2005, at 4, 18–49.

46. See, for example, NagelT Justice and nature. Oxford Journal of Legal Studies1997;17(2):303–21, at 315.

47. For discussion of the ways in which biological interventions might influence the moral status of our conduct, see DouglasT Enhancing moral conformity and enhancing moral worth. Neuroethics2014; 7: 75–91.2460048610.1007/s12152-013-9183-yPMC3933743

48. See, for a recent review, ThibautF, BarraFDL, GordonH, CosynsP, BradfordJMW The World Federation of Societies of Biological Psychiatry (WFSBP) guidelines for the biological treatment of paraphilias. World Journal of Biological Psychiatry2010;11(4):604–55.2045937010.3109/15622971003671628

49. BondAJ Antidepressant treatments and human aggression. European Journal of Pharmacology2005;526(1–3):218–25.1625323110.1016/j.ejphar.2005.09.033

50. KhanzodeL, SaxenaK, KraemerH, ChangK, SteinerH Efficacy profiles of psychopharmacology: Divalproex sodium in conduct disorder. Child Psychiatry & Human Development2006;37(1):55–64.1692717710.1007/s10578-006-0019-4

51. DanzigerS, LevavJ, Avnaim-PessoL Extraneous factors in judicial decisions. Proceedings of the National Academy of Sciences2011;108(17):6889–92.10.1073/pnas.1018033108PMC308404521482790

52. For discussion of various ways in which existing drugs may influence morally significant conduct, see Levy N, Douglas T, Kahane G, Terbeck S, Cowen P, Hewstone M, Savulescu J, Are you morally modified? The moral effects of widely used pharmaceuticals. *Philosophy, Psychiatry and Psychology*; Forthcoming.

53. CrockettMJ, Apergis-SchouteA, HerrmannB, LiebermanMD, MüllerU, RobbinsTW, et al Serotonin modulates striatal responses to fairness and retaliation in humans. Journal of Neuroscience2013;33(8):3505–13.2342667810.1523/JNEUROSCI.2761-12.2013PMC3593678

54. De JonghR, BoltI, SchermerM, OlivierB Botox for the brain: Enhancement of cognition, mood and pro-social behavior and blunting of unwanted memories. Neuroscience & Biobehavioral Reviews2008;32(4):760–76.1829588510.1016/j.neubiorev.2007.12.001

